# Enhanced diagnosis of terra firma-forme dermatosis by dermoscopy and line-field confocal optical coherence tomografphy

**DOI:** 10.1016/j.jdcr.2025.10.033

**Published:** 2025-10-28

**Authors:** Andrea Calogero Trecarichi, Maria Rita Nasca, Francesco Lacarrubba, Giuseppe Micali, Anna Elisa Verzì

**Affiliations:** Dermatology Clinic, University of Catania, Catania, Italy

**Keywords:** dermoscopy, LC-OCT, line-field confocal optical coherence tomography, pediatric, terra firma-forme

## Introduction

Terra firma-forme dermatosis (TFFD), also known as Duncan dirty dermatosis, is an idiopathic benign skin disease likely resulting from altered keratinocyte maturation.^1^ It has a bimodal peak of incidence with a first peak during the first 10 years of life and a second peak during 60 to 80 years of age. There is no gender predilection, and literature on genetic inheritance is inconclusive.^2^ The conditions’ average duration is a few months with a low recurrence rate.^3^ An association between TFFD and atopic dermatitis has been described in a pediatric population.^4^ Pathogenesis of TFFD is still unclear. Inhibition of the epidermal keratinoplastic activities, leading to impaired desquamation, corneocytes clumping, retention hyperkeratosis, and accumulation of sebum and dirt, has been considered.^1^^,^^5^^,^^6^ Histopathologic examination shows lamellar hyperkeratosis with intracorneal orthokeratotic whorls, keratotic plugging of follicular orifices, keratin globules in the stratum corneum, papillomatosis of the epidermis associated with irregular acanthosis, increased melanin pigment in the basal layer, and minimal lymphocytic liquefaction. In the dermis, edema, pigment-laden macrophages, perivascular lymphocytic infiltration, and erythrocyte extravasation may be observed.^6^^,^^7^ However, skin biopsies are seldom performed, and diagnosis is usually made based on clinical findings, with complete clearance of the typical polymorphous stuck-on appearing patches, mimicking dirty skin, after rubbing with 70% isopropyl alcohol.^2^^,^^3^ Interestingly, noninvasive techniques such as dermoscopy and reflectance confocal microscopy have also been used in an attempt to provide valuable additional information in cases where the diagnosis is uncertain.^2^^,^^7^, ^8^, ^9^

In this case report, we describe the features of a case of TFFD obtained by line-field confocal optical coherence tomography (LC-OCT), a recent noninvasive imaging tool that allows an “in vivo” visualization with a cellular resolution, of the epidermal layers until the superficial dermis with both horizontal and vertical views.^10^

## Case report

**Fig. 1:**
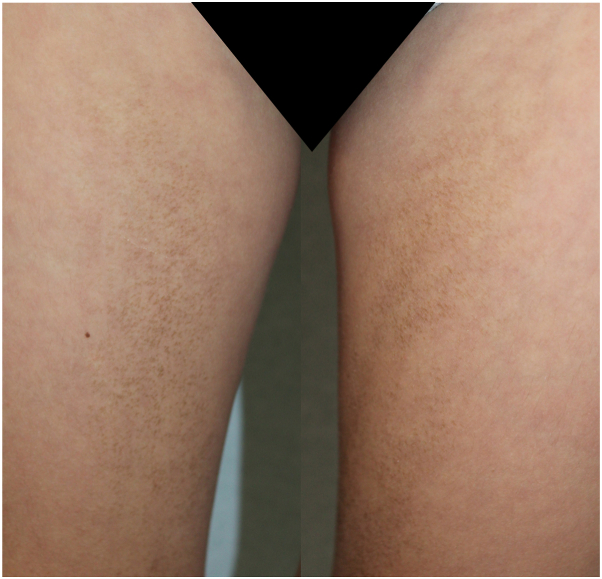
Terra firma-forme dermatosis: dirt-like’ stuck-on appearing patches and hyperpigmented papules located symmetrically on the right and left inner thighs.

A 10-year-old Caucasian boy presented with a 1-year history of asymptomatic, hyperpigmented, brownish macules scattered on his trunk and limbs. Family and past personal history were negative for any concomitant disease and only revealed an episode of glomerulonephritis at the age of 4 years, which resolved without sequelae. Any local trauma or inflammatory disease was excluded, as well as prior application on the affected body sites of topical agents other than emollients and keratolytics, ineffectively used to improve the condition after previous medical advice. Physical examination showed brownish, “dirt-like,” stuck-on appearing patches located symmetrically on the neck, armpits, abdomen, groin, and inner thighs with scattered islands of normal-appearing skin. On palpation, a smooth, velvety texture with coalescing micropapules arising from some hyperpigmented finely scaling patches could be appreciated (Fig 1).

**Fig. 2:**
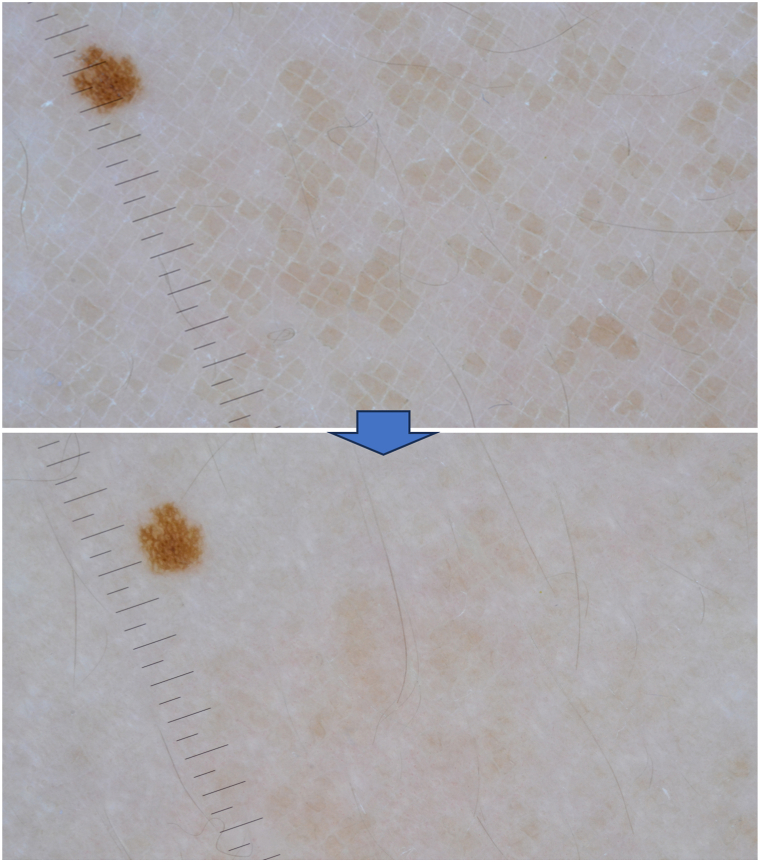
Terra firma-forme dermatosis: dermoscopy showing polygonal patches arranged in a mosaic and cobblestone pattern and their disappearance after rubbing with 70% isopropyl alcohol. The small nevus was used as a landmark. (Original magnification: ×10.)

**Fig. 3:**
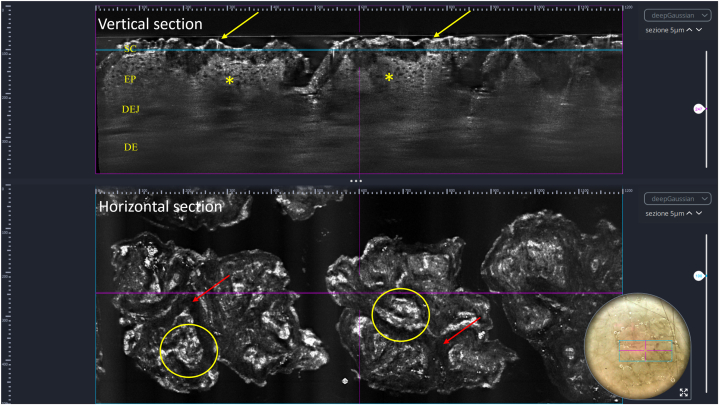
Terra firma-forme dermatosis: line-field confocal optical coherence tomography showing, at vertical view, hyperrefractive and thickened SC (*yellow arrows*) and irregular thickening of the granular–spinous layer (*yellow asterisks*); at horizontal view, hyperrefractive structures (*yellow circles*) interspersed with darker curvilinear areas (red arrows) are visible. The blue horizontal line in the vertical section corresponds to the level of the horizontal section. *DE*, Dermis; *DEJ*, dermoepidermal junction; *EP*, epidermis; *SC*, stratum corneum.

**Fig. 4:**
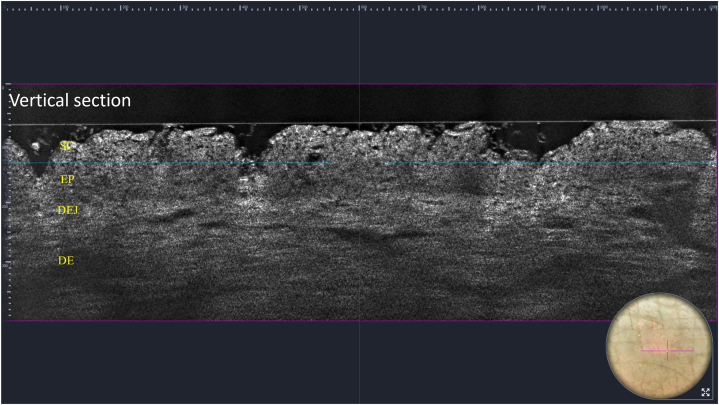
Terra firma-forme dermatosis: line-field confocal optical coherence tomography (vertical view) after rubbing with 70% isopropyl alcohol: normalization of the SC. *DE*, Dermis; *DEJ*, dermoepidermal junction; *EP*, epidermis; *SC*, stratum corneum.

Polarized dermoscopy (3Gen DermLite Foto II Pro) showed polygonal patches arranged in a mosaic and cobblestone pattern (Fig 2). LC-OCT (DeepLive, DAMAE Medical) highlighted a thickened hyperrefractive stratum corneum as well as irregular thickening of the granular–spinous layer in the vertical view (Fig 3). It also showed the presence of hyperrefractive structures interspersed with darker curvilinear areas in horizontal view (Fig 3). After rubbing with 70% isopropyl alcohol onto the affected areas, total clearing was observed both clinically and at dermoscopy (Fig 2), with LC-OCT showing a consistent reduction of the hyperkeratosis (Fig 4).

## Discussion

Recognition of TFFD is important, especially in the pediatric and adolescent age, because it can easily be misdiagnosed as dermatosis neglecta, a condition resulting from poor personal hygiene^8^ that may cause psychological discomfort and social isolation. Unlike TFFD, in which vigorous rubbing of the affected area with a gauze soaked in 70% isopropyl alcohol is necessary to detach the hyperpigmented corneous lamellae, in dermatosis neglecta, a simple resumption of proper hygiene with soap and water easily results in skin clearance.^8^ Differential diagnosis also includes postinflammatory hyperpigmentation, ichthyosis, acanthosis nigricans, confluent and reticulated papillomatosis, dirty neck syndrome, tinea versicolor, verrucous epidermal nevi, ashy dermatosis, and Dowling-Degos syndrome.

In our experience, LC-OCT, by providing both horizontal and vertical views, was of great help in the noninvasive diagnosis of TFFD: at vertical view, it showed a superficial hyperrefractive stratum corneum and an irregular thickening of the underlying epidermal layers that histopathologically correlate with the typical lamellar hyperkeratosis without parakeratosis and acanthosis, respectively; at horizontal view, it showed in the stratum corneum the presence of hyperrefractive structures interspersed with darker curvilinear areas corresponding to the typical intracorneal orthokeratotic whorls described at histopathology. LC-OCT also allowed for the detection of the disappearance of hyperkeratosis and intracorneal whorls with thinning of the stratum corneum after rubbing with 70% isopropyl alcohol, which is pathognomonic for a correct TFFD diagnosis. Thus, it should be considered a useful, real-time, and noninvasive additional tool for the diagnosis of TFFD.

The typical TFFD features observed at dermoscopy and reflectance confocal microscopy,^2^^,^^6^, ^7^, ^8^, ^9^ as well as our findings revealed by LC-OCT, are summarized in Table I, along with histopathologic correlations.

**Table I:** Diagnostic clues of terra firma-forme dermatosis at dermoscopy, reflectance confocal microscopy, and line-field confocal optical coherence tomography and their histopathologic correlation

Technique	Diagnostic clues	Histopathologic correlation
Dermoscopy	Brownish polygonal plate-like scales arranged in a mosaic or cobblestone pattern	Lamellar orthohyperkeratosis
	Linear and curvilinear structures (seborrheic keratosis-like pattern)	Intracorneal orthokeratotic whorls, underlying papillomatosis, irregular acanthosis
	Perifollicular hyperpigmentation	Basal layer hypermelanosis
RCM	Superficial hyperrefractive compact areas interspersed among dark serpiginous areas	Intracorneal keratin whorls
LC-OCT	Vertical view: hyperrefractive and thickened stratum corneum and irregular thickening of the granular–spinous layer	Lamellar hyperkeratosis (without parakeratosis) and acanthosis
	Horizontal view: presence of hyperrefractive structures interspersed with darker curvilinear areas at the corneal level	Intracorneal orthokeratotic whorls

*LC-OCT*, Line-field confocal optical coherence tomography; *RCM*, reflectance confocal microscopy.

In conclusion, TFFD is a readily diagnosable and treatable condition, albeit frequently misdiagnosed because of its similarity to other diseases. In cases where the diagnosis is uncertain, noninvasive diagnostic methods, such as dermoscopy and LC-OCT, may avoid the need to perform a biopsy.

## Conflicts of interest

None diclosed.

## References

[1] Duncan W.C., Tschen J.A., Knox J.M. (1987). Terra firma-forme dermatosis. Arch Dermatol.

[2] Mohta A., Sarkar R., Narayan R.V., Deoghare S., Arora A. (2023). Terra firma-forme dermatosis-more than just dirty. Indian Dermatol Online J.

[3] Sechi A., Patrizi A., Savoia F., Leuzzi M., Guglielmo A., Neri I. (2021). Terra firma-forme dermatosis: a systematic review. Int J Dermatol.

[4] Neri I., Savoia F., Tengattini V., Sechi A., Rucci P., Patrizi A. (2018). Terra firma-forme dermatosis is underestimated in children and is associated with atopic dermatitis. J Eur Acad Dermatol Venereol.

[5] Berk D.R., Bruckner A.L. (2011). Terra firma-forme dermatosis in a 4-month-old girl. Pediatr Dermatol.

[6] Erkek E., Sahin S., Çetin E.D., Sezer E. (2012). Terra firma-forme dermatosis. Indian J Dermatol Venereol Leprol.

[7] Lora V., Ardigò M., Catricalà C., Cota C. (2014). Terra firma-forme dermatosis. J Cutan Pathol.

[8] Errichetti E., Stinco G. (2017). Dermoscopy in terra firma-forme dermatosis and dermatosis neglecta. Int J Dermatol.

[9] Elmas Ö.F., Uyar B., Kilitçi A., Kutlu Ö. (2020). Dermoscopic patterns of terra firma-forme dermatosis. Dermatol Online J.

[10] Cappilli S., Paradisi A., Di Stefani A. (2024). Line-field confocal optical coherence tomography: a new skin imaging technique reproducing a "virtual biopsy" with evolving clinical applications in dermatology. Diagnostics (Basel).

